# Influence of luting cement on the biomechanical behavior of Bioflx crowns

**DOI:** 10.1186/s12903-025-06339-x

**Published:** 2025-06-21

**Authors:** Ahmed S. Waly, Salah A. Yousief, Mohamed Elsayed Moteea, Mohammad Said Abu Samadah, Mohamed Taha Elfezary

**Affiliations:** 1 Department of Pediatric Dentistry, Faculty of Dentistry, Al-Azhar University, Assiut, Egypt; 2 Department of Restorative and Prosthetic Dental Sciences, College of Dentistry, Dar Al Uloom University, Riyadh, 13313 Saudi Arabia; 3 Department of Crown and Bridge, Faculty of Dentistry, Al-Azhar University, Assiut, Egypt

**Keywords:** Finite element analysis, Bioflx crown, GIC cement, GIC modified with self-curing resin, Resin cement

## Abstract

**Background:**

The objective of this study was to examine the impact of various types of cements on primary molar tooth restored with a Bioflx crown.

**Methods:**

Three distinct finite element models were developed to represent three different cements; (1) conventional glass ionomer cement (GIC) (approximately 17 μm thick), (2) self-curing resin-modified GIC (RMGIC) (approximately 10 μm thick), and (3) self-cure resin cement (RC) (approximately 30 μm thick), all supporting/securing the Bioflx crown (approximately 330 microns thick). The geometry of the lower second primary molar was captured by laser scanning and then processed to create a solid model. This model was then imported into finite element software to assign materials, create a mesh, and evaluate stress and deformation under average normal occlusal loads. An applied load of 330 N was evaluated at three angles: vertical, oblique at 45°, and lateral.

**Results:**

The results indicated that model #2 (self-curing resin-modified GIC) exhibited the greatest deformation across all model components under the three loading conditions. The results for conventional GIC cement were comparable to those of self-cure resin cement. The resin-modified self-curing GIC (model #2) demonstrated high stress levels under lateral and oblique loads. Additionally, elevated stress concentrations were observed in the cortical bone region.

**Conclusions:**

A cement type with a higher modulus of elasticity may be preferred over other types, in addition to the potential for use with a thinner thickness. Therefore, conventional GIC demonstrated the best performance among the cements evaluated in this study. This was followed by self-cure resin cement, while self-curing resin-modified GIC might be excluded due to expectation of failure.

**a finite element analysis**.

**The full names of the authors**:

## Background

The American Academy of Pediatric Dentistry advocates for full coverage of a tooth affected by decay or one that has undergone vital pulp therapy or root canal treatment, assuming it is expected to remain functional for more than two years [1]. Stainless steel crowns (SSC) are preferred because of their versatility, ease of placement, affordability, and reliance on undercut for retaining the crown (active mechanical fit). However, the aesthetics of SSC may not be attractive to some children and their parents [2].

As the demand for pediatric aesthetic dentistry increases among parents, tooth-coloured zirconia crowns have become an visually attractive alternative [3]. Zirconia crowns offer excellent biocompatibility but necessitate more extensive tooth preparation compared to SSC and Bioflx crowns. Unlike SSC and Bioflx crowns, which rely on mechanical undercuts for retention, zirconia crowns require a passive fit, necessitating the removal of all undercuts to allow seating. Consequently, their retention depends primarily on luting cement rather than mechanical retention. This preparation technique is more invasive, requiring greater tooth reduction and more technique sensitive [4]. In pediatric patients, achieving this level of precision is particularly challenging due to limited cooperation and difficulties in maintaining a dry field for cementation, in addition to being more expensive [3, 5]. In contrast, SSC and Bioflx crowns require minimal tooth preparation, are less reliant on cement, and allow for a quicker, less invasive procedure and lower patient compliance demands.

Pediatric dentists have long sought a crown material that combines the attributes of stainless steel with the natural tooth color of zirconia. This led to the invention of a new type of crown, Bioflx, which integrates these two features and includes a snap fit. Bioflx crowns, a recent innovation in pediatric dentistry, are recognized for their flexibility and adaptability, combining the characteristics of stainless steel and zirconia crowns [6].

Bioflx crowns are constructed from a biocompatible hybrid resin polymer, designed to address issues related to ductility, color stability, and durability. Importantly, Bioflx crowns provide a flexible fit adaptation to the anatomic cervical convexity of primary teeth and are cemented with conventional GIC or self-curing RMGIC, with the added advantage of a more aesthetic appearance and conservative tooth preparation. However, there are currently few comprehensive studies that assess the properties of Bioflx crowns and their impact on clinical outcomes. In routine dental care, crown decementation is a source of annoyance for dentists, parents, and children alike. It leads to additional appointments, increased expenses, and the potential hazard of inadvertent crowning [7, 8].

Initial case reports and clinical trials indicate promising results in terms of ease of placement, aesthetic appeal, and clinical performance [9, 10]. In the inaugural clinical evaluation that examined the efficacy of the BioFlx crown secured with a conventional GIC, it was observed that retention was significantly lower compared to SSC [10].

Type and thickness of luting cement play a crucial role in stress absorption, distribution thus crown retention, the nature and magnitude of stress development in luting cement depends greatly on the formulation and film thicknesses, with the thicker layer causing faster stress development in glass-ionomer. The film thickness of luting cement varies from 10 to 30 μm [11]. The manufacturer of Bioflx crowns recommends using conventional GIC and self-cure RMGIC as luting agents for cementation. However, they do not provide specific recommendations regarding the cement thickness, clinicians should aim to follow best practices for cementation by ensuring the crown is well-fitted and excess cement is minimized.

The optimal cementation technique for Bioflx crowns remains unestablished, with no definitive evidence supporting an alternative approach to the manufacturer’s guidelines. Additionally, there is currently no available data on the most effective clinical protocol for cementing Bioflx crowns in pediatric dentistry. This study aims to evaluate the biomechanical performance of Bioflx crowns cemented with three different luting agents using finite element analysis.

### Objective

To evaluate the effect of different luting cements on the stress distribution and biomechanical behavior of Bioflx crowns using finite element analysis.

### Null hypothesis

There is no significant difference in the biomechanical performance of Bioflx crowns cemented with different luting agents.

## Methods

**Fig. 1:**
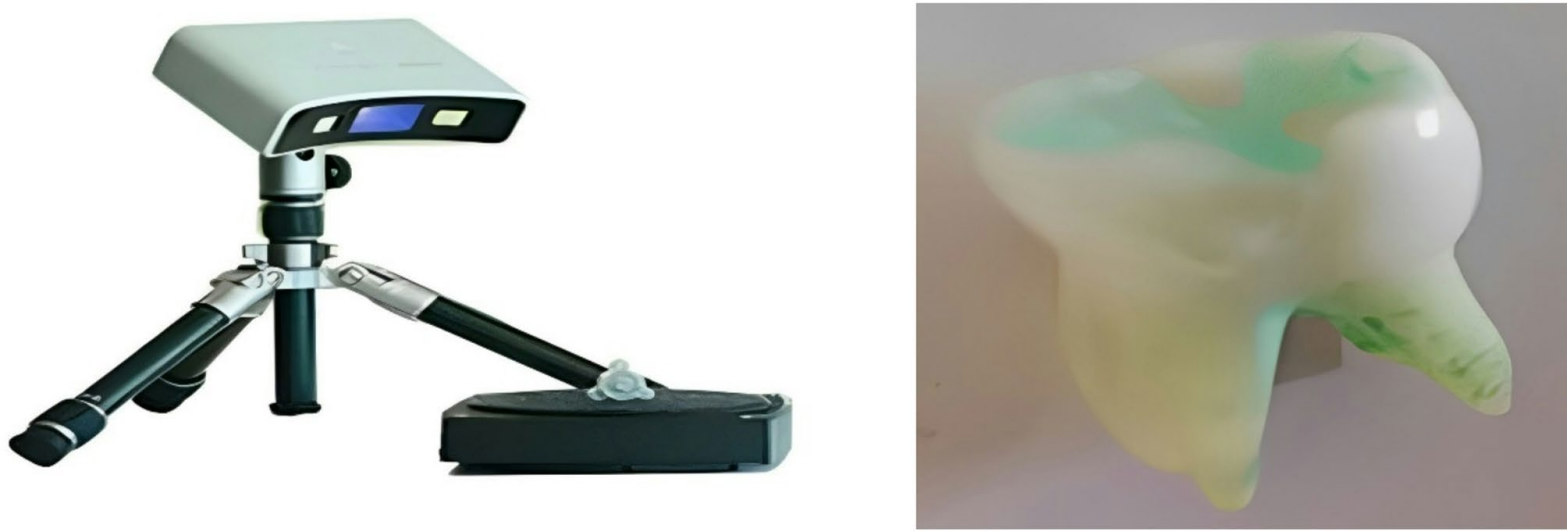
Laser scanner and scant tooth

For this study, three finite element models of the second primary mandibular molar were developed. The process began with laser scanning of a freshly extracted second primary mandibular molar, which had been removed due to periodontal disease (Aggressive Periodontitis), following parental consent [12]. The external geometry of the tooth was captured using a Geomagic Capture 3D Systems laser scanner (Cary, NC, USA), with an accuracy of approximately 30 microns (0.030 mm), as shown in Fig. 1 [12]. The scanned data was processed in STL format, which is compatible with various CAD and FEA software for further analysis. Intermediate software (Rhino 3.0 - McNeel Inc., Seattle, WA, USA) was used to trim the newly created surfaces using the acquired points. Since the scanner only captures external geometry, dentin was not directly scanned but was instead modeled separately based on standard anatomical references to ensure realistic structural representation. Cementum was also not explicitly included in the model due to its thin structure and minimal biomechanical influence on stress distribution. Each tooth component was modeled based on its unique anatomical and material characteristics. The enamel, the outermost layer of the tooth, was modeled with a thickness of 0.2 mm. Dentin was designed with a thickness of 2 mm, while the pulp, periodontal ligament, and crack design were not included in the model. The geometries were then exported to finite element analysis (FEA) software in STEP file format [13]. The material properties of enamel and dentin were assigned based on published mechanical data, including Young’s modulus and Poisson’s ratio (see Table 1 for detailed values).

Cortical and cancellous bone models were developed using the commercial computer-aided design software ‘SolidWorks’ version 14 (Dassault Systems SolidWorks Corp., France). Bone geometry was simplified and represented as two coaxial cylinders. The inner cylinder, with a diameter of 14 mm and a height of 22 mm, represented the cancellous bone and filled the inner space of the outer cylinder, which had a shell thickness of 1 mm and represented the cortical bone (with an outer diameter of 16 mm and a height of 24 mm) [14, 15].

Thin structures such as the Bioflx crown (approximately 330 μm) and cement layers of 17, 10, and 30 μm thicknesses were created using a series of Boolean operations in the ANSYS environment (ANSYS Inc., Canonsburg, PA, USA). These operations were performed to finalize the models being evaluated. All materials entered into ANSYS were considered homogenous, isotropic, linear, and elastic, as listed in Table 1.

**Table 1:** Material properties of the finite element model (s) [15, 16]

Material	Young’s modules [GPa]	Posison’s ratio
**Bioflx Crown** [15]	5,030	0.39
**Cement** [16]**: GIC**	16,900	0.30
RMGIC	4,000	0.30
Resin Cement	7,000	0.27
**Pediatric Dental Hard Tissues and Bone** [15]**: Enamel**	80,350	0.33
Dentine	19,890	0.31
Cortical bone	14,700	0.30
Cancellous bone	0.490	0.30

**Fig. 2:**
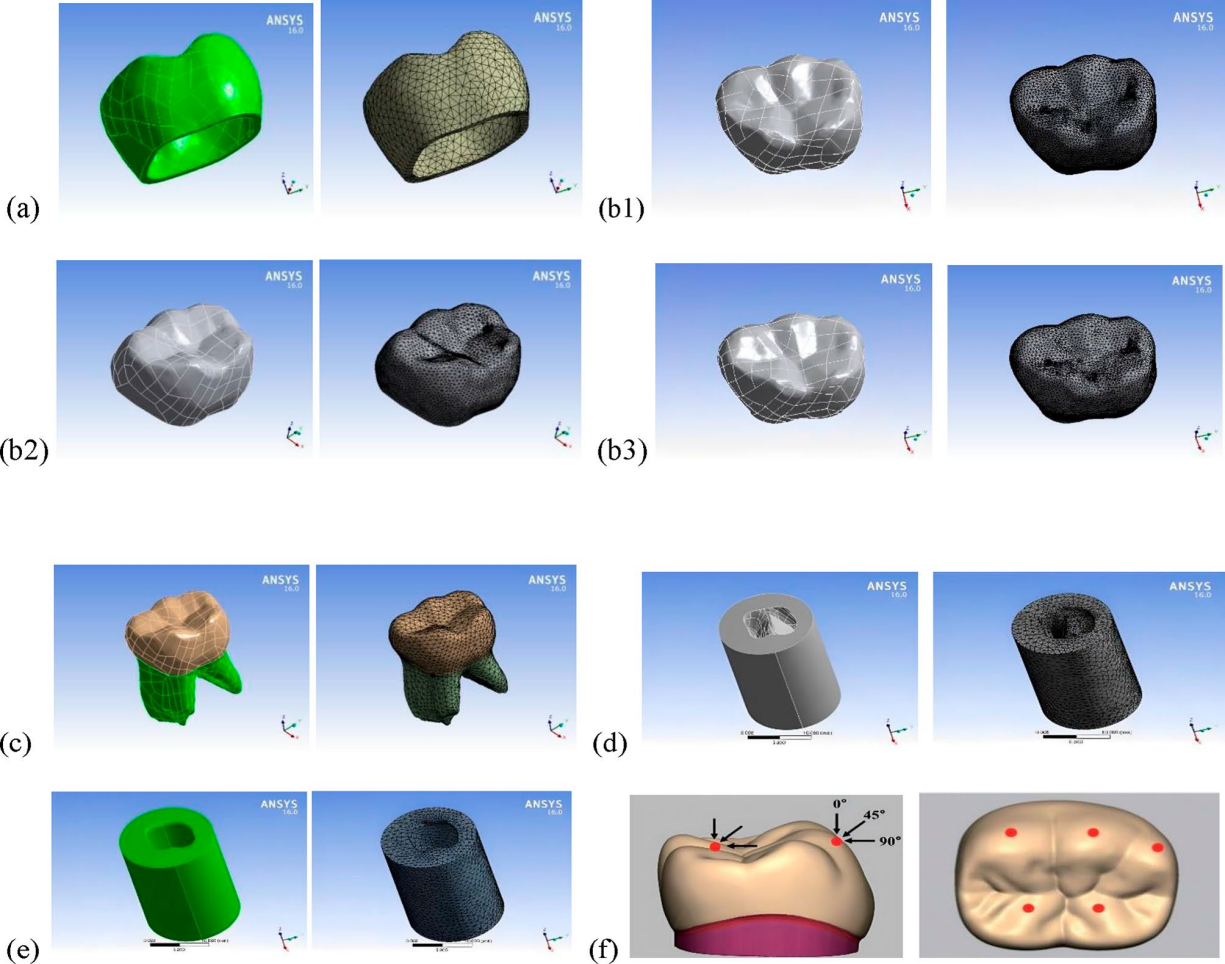
Screen shots for model components and its mesh (**a**) Bioflx-crown, (**b**1-3) sample of the three cement layers, (**c**) tooth structure, (**d**) cancellous bone, (**e**) cortical bone, and (**f)** Loading points

The components of the models were glued together using a solid 3D brick element, specifically 187, which has three degrees of freedom (allowing translation along the primary axes) [12]. The subsequent quantities of nodes and elements are enumerated in Table 2. Visual representations of the components of the meshed model can be found in Fig. 2, captured as ANSYS screenshots.

**Table 2:** Mesh density of the two models’ components

	Model 1:	GIC-17 μm	Model 2:	RMGIC-10 μm	Model 3:	RC − 30 μm
Material	Number of elements	Number of nodes	Number of elements	Number of Nodes	Number of elements	Number of Nodes
**Bioflx Crown**	9,159	16,086	7,873	14,360	9,924	17,119
**Cement layer**	24,835	49,702	49,669	79,414	20,198	40,566
**Dentine**	34,751	50,378	34,802	50,455	34,802	50,455
**Enamel**	7,286	13,287	7,286	13,287	7,286	13,287
**Cortical bone**	11,087	21,644	11,087	21,644	11,087	21,644
**Cancellous bone**	72,975	100,603	66,677	94,057	75,803	106,329

Each model was subjected to three different loading scenarios: a vertical load of 330 N, an oblique load at 45o and a lateral load. The load was applied at three points on the outer slopes of the buccal cusps and two points on the inner slopes of the lingual cusps (see Fig. 2.f) [12, 14]. Before extraction of analysis results, the models were verified against comparable studies [3, 12].

As a boundary condition, the lowest plane of the model was considered fixed in all three directions. Using an HP Z820 Workstation equipped with dual Intel Xeon E5-2660, 2.2 GHz processors, and 64GB RAM, nine linear static analyzes were executed.

## Results

The analyzes yielded numerous graphical distributions for deformations and stresses. These distributions highlighted critical locations that could potentially exhibit high stress, significant deformation, or failure. Comparisons between the components of the three models (each model representing a different type of cement) under identical loading conditions led to insightful conclusions. The distributions of stresses and deformations revealed negligible or minor alterations in critical locations.

**Fig. 3:**
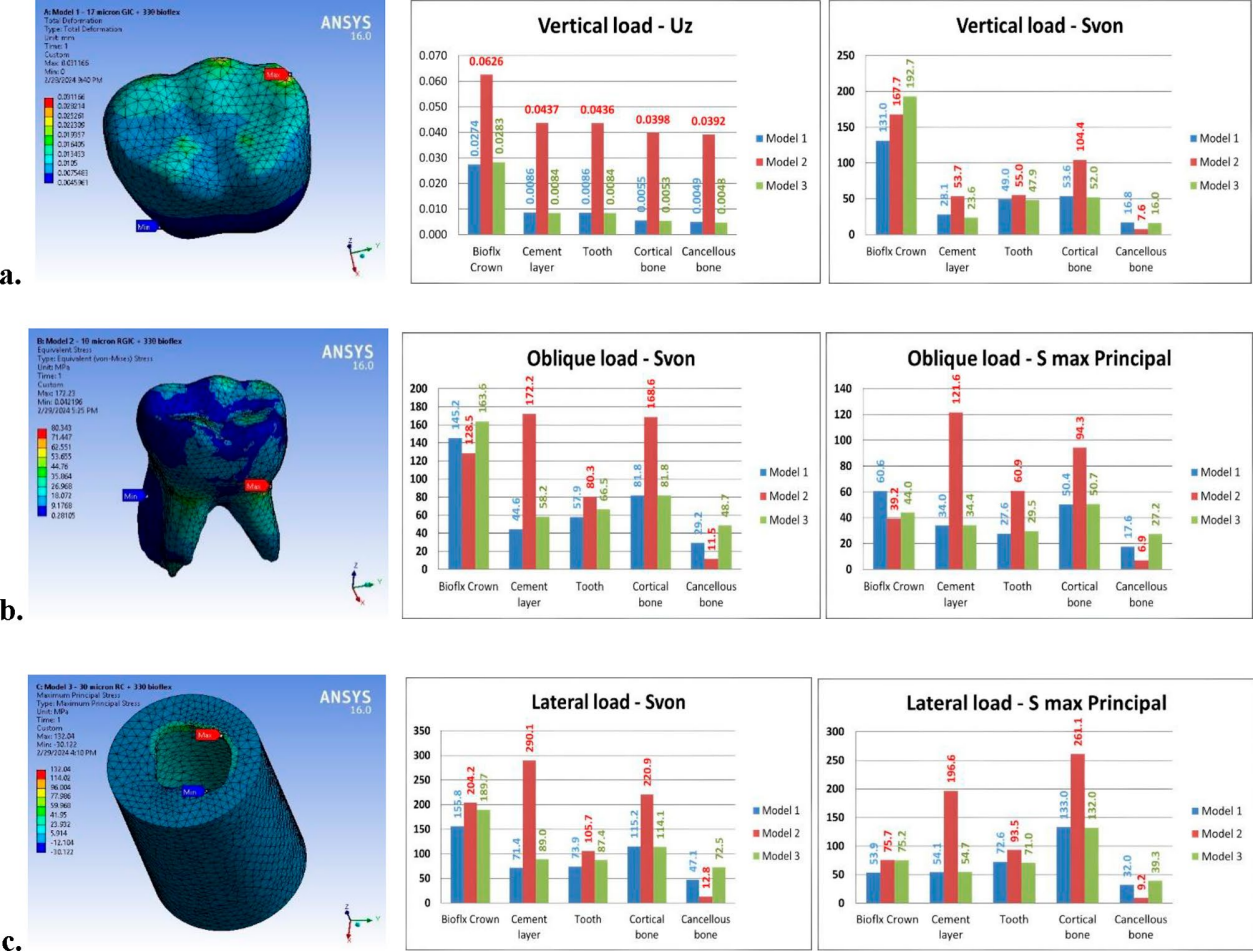
Sample results and comparisons; (**a**) model #1 crown total deformation distribution, vertical deformation, and Von Mises stress values comparison among the three models, (**b**) model #2 tooth structure Von Mises stress distribution, Von Mises stress, and maximum principal stress values comparison among the three models, (**c**) model #3 cortical bone maximum principal stress distribution, Von Mises stress, and maximum principal stress values comparison among the three models

Figure 3.a illustrates the total deflection of the Bioflx crown body in Model #1 (positioned above a 17 μm conventional GIC cement layer) when subjected to vertical loading. In addition, it compares the extreme values of vertical deformation, and Von Mises stress observed in each component of the three models. These comparisons suggested that the use of a self-cure RMGIC cement layer of approximately 10 μm (Model #2) could result in a vertical deformation six times greater than that observed with other models. On the contrary, the first and third models exhibited similar deformation and stress values, although the third model had a cement layer thickness double that of the first model (30 μm self-cure RC compared to 17 μm conventional GIC cement). Most of the deformation was recorded in the cortical bone (tooth support), with values of around 5 and 40 μm for Models #1 & #3 and Model #2, respectively. The net deformation of the tooth structure, the cement layer and the Bioflx crown was approximately 1 μm or less, showing safe values and reducing the possibility of failure. The most extreme deformation under vertical loading was observed at the tip of the cusp, directly below the loading site.

Under vertical loading, the stresses on the tooth and bone showed similar and moderate levels, except for the cortical bone in Model 2, which showed a value twice that of the other two models. Variation in Von Mises stress values on the cement layer and Bioflx crown across the three models could suggest the superiority of one type of cement over the others.

Model #3, which used RC layer of approximately 30 μm, experienced the lowest stress value, but induced the highest stress in the Bioflx crown (approximately 193 MPa). The conventional GIC cement layer (Model #1) endured approximately 25% more stress compared to self-cure RC but resulted in the lowest stress on the Bioflx crown (approximately 130 MPa). Although the self-curing RMGIC cement layer received a Von Mises stress value double that of the other two cement types, the stress values observed on the Bioflx crown in Model #2 were moderate, falling between the values of the other two models.

Figure 3.b shows the Von Mises stress distribution on the tooth structure under oblique loading in Model #2, which uses a 10 μm self-cure RMGIC cement layer. The highest stress values were observed at the connection of the tooth and the cortical bone. Under oblique loading, Model #2 showed higher stress levels than the other two models, except for the crown body and the spongy bone. Although Von Mises stress is more severe than the largest principal stress, both criteria suggest that the self-curing RMGIC cement layer (Model #2) would fail under oblique loading, while the other types might withstand the load. The underlying structures (tooth and bone) may survive within the three models, except for the cortical bone in Model #2, which reaches a critical stress level. On the contrary, Model #1 proved equivalent or lower stresses than Model #3 under oblique loads on cement layers and all natural tissues.

Figure 3.c presents the maximum distribution of tensile stress in the cortical bone under lateral loading in Model #3, which employs a 30 μm self-cure RC layer. The highest stress values were found at the bone-cortical tooth connection. Once again, under lateral loading, Model #2 showed higher stress levels than the other two models, except in the spongy bone only. It is certain that the cement layer and cortical bone in Model #2 will fail under lateral load due to extremely high stresses, while the cortical bone may reach critical stress values in the other two models. However, model #1 showed slightly better performance (less stress) than model #3.

## Discussion

The objective of this study was to evaluate the influence of various luting cements on the stress distribution in primary molars restored with BioFlx crowns. The cements selected conventional GIC, self-curing RMGIC, and self-cure RC were chosen based on their clinical relevance in paediatric dentistry. The null hypothesis was that cement type would have no effect on stress patterns. Our FEA results demonstrated significant differences among cements, so the null hypothesis is rejected, indicating that cement selection does affect the biomechanical behavior of the restoration.

### Conventional glass ionomer cement (GIC)

GIC is widely used in pediatric restorations due to its biocompatibility, fluoride release, and chemical adhesion, which enhances retention and minimises microleakage [17]. Its thermal expansion coefficient closely matches that of natural tooth structures, reducing the risk of marginal gaps [18]. Furthermore, its mechanical properties align well with dental tissues, contributing to stress distribution and reducing failure risks [17].

### Resin-Modified glass ionomer cement (RMGIC)

RMGIC combines the benefits of conventional GIC with improved adhesive properties and sustained fluoride release, offering enhanced retention and caries resistance [12]. Its adaptability simplifies clinical procedures while ensuring a durable and preventive restoration outcome, making it particularly suitable for pediatric applications.

### Self-Cure resin cement (RC)

Resin cement is preferred for its superior adhesion, enhancing crown retention and longevity, especially in pediatric patients with high occlusal forces. FEA studies have demonstrated its favorable stress distribution patterns under pediatric stainless-steel crowns, supporting its potential for improved durability [19, 20]. Despite its increased thickness, its lower modulus of elasticity may contribute to flexibility underload.

Among the three cements, conventional GIC exhibited properties most closely resembling natural dental tissues, ensuring optimal stress distribution and structural integrity. Its fluoride release and ease of application make it a cost-effective option, particularly for high-caries-risk populations [21, 22] – [23]. The balance of its mechanical characteristics with those of dentine and cortical bones further supports its clinical suitability.

### Influence of cement layer thickness

The cement layer is critical for restoration stability, influencing stress distribution and longevity. A thicker cement layer can improve performance by enhancing the cement’s lifespan and reducing stress transmission to the surrounding bone [24, 25]. In pediatric patients, where bones are less rigid than in adults, managing stress transfer is essential to preventing microfractures and structural compromise.

Our findings underscore the importance of selecting a cement that aligns with the biomechanics of the oral environment. Given its favorable properties, conventional GIC remains a reliable choice for pediatric Bioflx crowns, offering a balance between durability, retention, and stress distribution.

Our study investigated three types of cement: conventional GIC, self-curing RMGIC, and RC. Interestingly, we saw that the thicker cement layer (specifically, the resin cement in model #3) showed a response comparable to that of moderately thick conventional GIC cement. Despite its greater thickness, the resin cement showed equivalent behavior under load, suggesting that it could be a practical alternative.

In particular, the modulus of elasticity of the thicker cement layer (resin cement) was half that of the modulus of elasticity of the conventional GIC. This finding highlights an essential trade-off: While a thicker cement layer may improve performance, its lower modulus of elasticity could affect overall stiffness and load distribution.

Scientifically, the concept of cement layer thickness and its impact on restoration longevity and reduction of bone stress have been explored in earlier research. Furthermore, studies on the biomechanics of bone-cement interfaces emphasize the importance of cement properties to maintain the stability of the prosthesis [26, 27].

Primary first and second molars are typically lost by around 9–12 years of age, meaning these teeth have a limited functional period. In this context, the significant stress differences observed among cements may not translate into markedly different clinical outcomes before natural exfoliation. All tested cements might adequately serve the relatively short lifespan of primary crowns. Nevertheless, choosing a cement that produces lower stress concentrations could still be beneficial to maximize crown retention and durability during the primary tooth’s service life.

Limitation.

It should be noted that our FEA model incorporates simplifying assumptions. Materials were modeled as homogeneous, isotropic, and linearly elastic, and only static occlusal loads were applied. Such assumptions mean that the model may not fully replicate in vivo conditions. Consequently, the numerical results should be interpreted with caution, and further clinical or experimental work is needed to validate these predictive findings.

In conclusion, this FEA study demonstrated that the type of luting cement significantly influences the stress distribution in pediatric BioFlx crown restorations. The null hypothesis of no difference among cements is therefore rejected. These findings directly meet the study’s objective by confirming that cement choice affects the biomechanical performance of the restoration. Among the cements evaluated, conventional GIC cement yielded the most favorable stress profile, suggesting it may be a preferred choice for enhancing crown durability in primary molars. Future clinical studies are needed to confirm these implications.

## Data Availability

All data generated or analyzed during this study are included in this published article and the corresponding author can provide the data upon request.

## Abbreviations

GIC: Glass Ionomer Cement
RMGIC: Resin Modified Glass Ionomer Cement
RC: Resin Cement
SSC: Stainless Steel Crown
FEA: Finite Element Analysis
